# Interindividual Differences in Mid-Adolescents in Error Monitoring and Post-Error Adjustment

**DOI:** 10.1371/journal.pone.0088957

**Published:** 2014-02-18

**Authors:** Sarah Rodehacke, Eva Mennigen, Kathrin U. Müller, Stephan Ripke, Mark J. Jacob, Thomas Hübner, Dirk H. K. Schmidt, Thomas Goschke, Michael N. Smolka

**Affiliations:** 1 Neuroimaging Center, Technische Universität Dresden, Dresden, Germany; 2 Department of Psychiatry and Psychotherapy, Technische Universität Dresden, Dresden, Germany; 3 Institute of General Psychology, Biopsychology and Methods of Psychology, Department of Psychology, Technische Universität Dresden, Dresden, Germany; Centre Hospitalier Universitaire Vaudois and University of Lausanne, Switzerland

## Abstract

A number of studies have concluded that cognitive control is not fully established until late adolescence. The precise differences in brain function between adults and adolescents with respect to cognitive control, however, remain unclear. To address this issue, we conducted a study in which 185 adolescents (mean age (SD) 14.6 (0.3) years) and 28 adults (mean age (SD) 25.2 (6.3) years) performed a single task that included both a stimulus-response (S-R) interference component and a task-switching component. Behavioural responses (i.e. reaction time, RT; error rate, ER) and brain activity during correct, error and post-error trials, detected by functional magnetic resonance imaging (fMRI), were measured. Behaviourally, RT and ER were significantly higher in incongruent than in congruent trials and in switch than in repeat trials. The two groups did not differ in RT during correct trials, but adolescents had a significantly higher ER than adults. In line with similar RTs, brain responses during correct trials did not differ between groups, indicating that adolescents and adults engage the same cognitive control network to successfully overcome S-R interference or task switches. Interestingly, adolescents with stronger brain activation in the bilateral insulae during error trials and in fronto-parietal regions of the cognitive control network during post-error trials did have lower ERs. This indicates that those mid-adolescents who commit fewer errors are better at monitoring their performance, and after detecting errors are more capable of flexibly allocating further cognitive control resources. Although we did not detect a convincing neural correlate of the observed behavioural differences between adolescents and adults, the revealed interindividual differences in adolescents might at least in part be due to brain development.

## Introduction

Cognitive control denotes several functions within the cognitive system that are necessary for performing non-routine tasks or coping with challenging situations, e.g. for shifting flexibly between competing tasks. Flexibility is needed in adaptive self-regulation and goal-directed behaviour [1], [2] or “when prepotent tendencies have to be overcome” [3]. Previous research focused on, amongst others, two phenomena of cognitive control: effects of interference and task-switching effects [4], [5]. The former arises from interference on the level of stimulus-response (S-R) [3], the latter from overcoming the previous task sets [6]–[8].

The conflict-monitoring hypothesis [9] proposes that the so-called “conflict-monitoring system” detects the occurrence of conflicts. This has been confirmed in studies with adults on the behavioural level [10], [11] and on the imaging level: Brain areas which are more activated during “conflict monitoring” include the anterior cingulate cortex (ACC) [5],[12]–[14] and the lateral prefrontal cortex (lPFC) [5], [15]. ACC and lPFC are also suggested to be involved in task switching [5], [16], [17]. In addition, results of a meta-analysis [16] provide strong evidence for the involvement of the pre-supplementary motor area (pre-SMA) and the inferior frontal junction in task-switching paradigms and in Stroop tasks. Furthermore, the most consistently observed brain region involved in task-switching paradigms seems to be the posterior parietal cortex (PPC) [18]–[20]. All of the above-mentioned brain areas constitute the so-called “cognitive control network” [21].

Parts of this network, especially the frontal regions, mature structurally throughout adolescence [22]. In line with structural development, higher cognitive functions also develop during this age period [23]. For a review regarding structural and functional brain development see Paus [24]. Bunge and colleagues [25] suggested that neural function changes considerably between the ages of 12 and 19 years. This functional development is confirmed by several studies showing that adolescents react as fast as adults, but make more mistakes in cognitive control tasks [26], [27]. In contrast, differences concerning neural activation during cognitive control tasks are not well understood. Neuroimaging studies regarding the development of cognitive control have so far yielded inconsistent findings: Within the ACC, in particular, prior studies showed an activation increase with age [27], [28] or a decrease with age [29] or even no differences in group comparisons between adolescents and adults in trials in which the participants answered correctly [26]. These contrary findings might mainly be due to the use of diverse tasks that assess different components of cognitive control as well as the use of different methods of analysis. Further, some studies controlled for performance [26], [27] while others did not [28], [29]. However, controlling for performance is important [30], especially as previous studies confirmed that there are differences on the behavioural level between adolescents and adults [26], [27]. Differences in the above mentioned studies were found in the ACC [27]–[29], in the inferior frontal gyrus [29], in the middle frontal gyrus (MFG) [28], [31] and in the superior frontal gyrus [28] as well in the PFC, in the insula and in parietal regions [27].

Like brain response in the cognitive control network error processing has been associated with activity in the ACC, anterior insula, parietal lobe, medial temporal lobe, basal ganglia and in the thalamus [32], [33]. Brain regions involved in neural post-error adjustments are also areas involved in cognitive control, especially the left anterior PFC as well as the right inferior parietal lobule [34]. The few neuroimaging studies that have been carried out to compare adolescents and adults regarding error processing also reported inconsistent findings: Prior studies reported weaker brain response in adolescents compared to adults in several parietal and frontal gyri (but not in the ACC) [35], in the left anterior insula and in the left basal ganglia [26] and in the rostral ACC [36]. Further, Velanova et al. [37] showed that activation differences between error and correct trials increase with age in the dorsal ACC. On the behavioural level previous studies [26], [37] revealed that adolescents committed significantly more errors than adults except for one study [35]. Again, this might mainly be due to the fact that different tasks and different methods of analysis were used. To sum up, besides behavioural differences, there also seem to be differences between adolescents and adults in brain response in correct and error trials, especially in frontal and parietal areas, during interference or switching paradigms.

An additional limitation of studies that have addressed cognitive control as well as error monitoring is that broad age ranges for adolescent subgroups were used, although it is well known that the brain structure develops considerably throughout adolescence [22], [24], [38]–[40].

From recent studies [26], [27] we conclude that on the behavioural level adolescents’ cognitive control is not as fully mature as in adults, as indicated by higher ER in adolescents. Although there are inconsistent findings concerning neural cognitive control and error processing, most studies concerning interference or switching paradigms [26]–[28] reported weaker brain response in adolescents in areas of the cognitive control network. Thus, we hypothesized that 1) adolescents would make more mistakes in our combined interference and switch task than adults, 2) adolescents would exhibit weaker brain responses than adults in neural systems involved in cognitive control (ACC, lPFC, pre-SMA, and PPC), and 3) there would be differences in neural error and post-error processing between adolescents and adults.

As higher cognitive functions develop throughout adolescence [23], we focus in this study on an age-homogeneous sample of mid-adolescents and compare them to adults using a combined interference and switch task. To our knowledge, no developmental study has implemented a task that combined interference and task switching elements in one paradigm. Such a task should boost the behavioural costs (i.e. RT, ER) associated with the two control demands [41]. Since strong control demands should increase the power for uncovering developmental effects, this paradigm should be able to broaden our knowledge of the development of cognitive control functions and error processing.

We collected data from a large adolescent sample (237 participants who, with the exception of seven adolescents, were 14 years old) and from 29 adults (mean age 25 years). We compared the two groups’ behaviour (RT and ER) and brain response (fMRI blood oxygen level-dependent (BOLD) response) during correct trials, during error trials and during correct trials after an error occurred or no response was given.

## Materials and Methods

### Subjects

Data were collected within the project “The adolescent brain”, which is funded by the German Federal Ministry of Education and Research (BMBF). A total of 260 adolescents took part in the study, of which 237 (mean age 14.6 years, only 7 adolescents were 13 or 15 years old) performed the interference and switch task. 29 adults (mean age 25.2 years) also performed the task.

All potential participants were screened for several exclusion criteria: presence of neurological disorders, current drug treatment, surgeries on heart or head and conditions posing safety issues for the MRI scan. Subjects participated in the study after giving written informed consent and for those who were under eighteen years old, at least one parent had to additionally agree to their participation by signing the consent form. The study was approved by the local research ethics committee (Ethics Committee of the Technische Universität Dresden) and conducted in accordance with the Declaration of Helsinki. All subjects were also screened for mental disorders using DAWBA – Developmental and Well-Being assessment [42] for adolescents, and CIDI – Composite International Diagnostic Interview [43] for adults. Subjects had normal or corrected to normal vision.

Table 1 gives an overview of age, gender, and number of excluded subjects in both samples. The final sample size was 185 adolescents (91 males) and 28 adults (16 males). Adolescents were recruited at local schools. Adult participants were students and staff members of the university.

**Table 1:** Details of the adolescent and adult sample.

		Adolescents	Adults
Age	Range	13.7–15.5	20–50
	Mean [SD]	14.6 [0.3]	25.2 [6.3]
Gender	Male	125	16
	Female	112	13
Number of subjects that performed the task		237	29
Exclusion criteria	At least one condition with less than 50% of all trials*	11	0
	Excessive movement (>3 mm volume to volume)	18	1
	Technical problems	14	0
	Normalization failed	5	0
	ADHD	3	0
	Hydrocephalus	1	0
Final sample size		185	28

*see *Behavioural data analysis*.

The imbalance in sample size between the adolescent and adult groups is due to the fact that there were limited resources for data collection from adults for a cross-sectional analysis. The main source of funding for this investigation came from the project “The adolescent brain”, which focuses on the assessment of longitudinal development during adolescence. The herein reported adolescent sample will be measured again at the ages of 16 and 18 using the same investigational tools.

### The Interference and Switch Task

**Figure 1:**
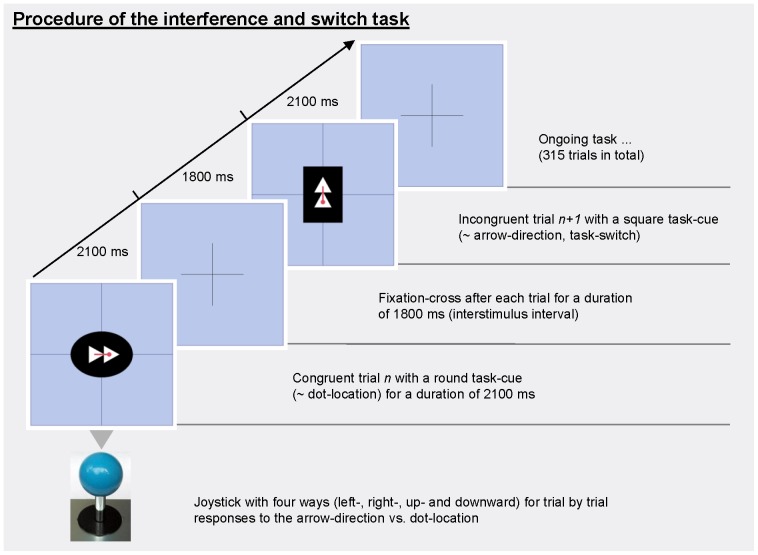
Procedure of the interference and switch task with two out of 16 possible stimuli.

We used event-related fMRI and employed a task that included both an S-R interference component and a task switching component. Our task is not a classical interference task (like Stroop) as no dominant response is primed: Both components were equally frequent. But, there is a relevant and an irrelevant task dimension leading to interference by overlapping S-R-mappings. In each trial, subjects were shown an arrow consisting of two touching triangles pointing in one of four (left, right, up or down) directions and a red dot located either at the tip or the tail of the arrow (see Figure 1). Subjects were instructed to move a joystick in the direction indicated by the arrow or the dot. The shape of the background served as a task cue: If the background was rectangular, subjects had to move the joystick in the direction of the arrow and ignore the position of the dot; conversely, if the background was circular, subjects had to respond to the position of the dot while ignoring the arrow direction. Stimuli could be congruent, i.e. dot and arrow were pointing in the same direction, or incongruent, i.e. the dot and the arrow were pointing in opposite directions. Hence, there were 8 stimuli with a rectangular and 8 with a circular background (in each case 1 congruent and 1 incongruent stimulus in each of the 4 possible directions). Stimuli were presented for 2.1 seconds, and the inter-stimulus interval with a fixation cross lasted 1.8 seconds. If subjects did not react within 2.1 seconds after stimulus onset, the trial was counted as a ‘missing’ trial. An example of a congruent and an incongruent stimulus as well as a task sequence is depicted in Figure 1.

Our task allowed us to set up a design with 2 within-subject factors of interest. The first factor represented the task transition (repetition vs. switch), and the second the congruence of the present trial (congruent vs. incongruent). Further, there were two within-subject factors of no interest, namely task (arrow vs. dot) to create the switch effect, and previous trial congruence (congruent vs. incongruent). Hence, there were four different conditions, i.e. repeat-congruent (rp_C), repeat-incongruent (rp_I), switch-congruent (sw_C), and switch-incongruent (sw_I). These were balanced with 64 trials each, meaning that 50% of all the trials were congruent and 50% incongruent, while 50% were repeat trials and 50% switch trials. Trials were presented in a randomised order. In order to avoid priming effects, there were no consecutive identical trials [44]. Trials were considered identical if they had the same task, the same congruency and the same direction of the dot and the arrow.

Outside the scanner, 35 trials of the interference and switch task were practised in a sitting position in front of a computer monitor. In addition, subjects performed another 35 practice trials in the scanner prior to scanning, to familiarize themselves with using the joystick while lying in the scanner.

Subjects were free to ask questions until imaging started. A total of 273 trials were performed. We included seven 20-second breaks in order to obtain a baseline measure for event-related fMRI and to give subjects time to relax and then to re-focus on the task. During a break, subjects were instructed to look at two parallel lines in the middle of the screen.

The first two trials and the last trial of the entire run were not included in the analysis. Moreover, the first two trials following a break were also discarded. Hence, there were 256 experimental trials.

### Behavioural Data Analysis

Behavioural data analysis included mean RT and ER. Trials in which subjects made an error or did not react within the given time window of 2.1 seconds after stimulus onset (so-called missing trials) were discarded from the RT analyses. Missing trials were very uncommon (mean omission rate for adolescents: 1.27% (SD 3.10%), and for adults: 0.25% (SD 0.85%)). Moreover, correct trials following error and missing trials were also discarded to disentangle task effects and post-error/post-missing effects, because subjects often react more slowly (so-called “post-error slowing”) and more accurately after an error [34], [45], [46]. Mean RTs were calculated for the remaining correct trials separately in the four experimental conditions, as well as for correct, error and post-error/post-missing trials, regardless of condition. Subjects were excluded if one of the four conditions contained less than 50% correct trials (i.e. less than 32 trials per condition). This exclusion criterion applied to 11 adolescent subjects (see Table 1). Further, ERs were calculated for every condition:
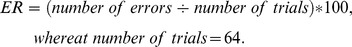



Values were calculated with Matlab 7.5 (MathWorks Company, Natick, MA, USA). Further calculations were processed with SPSS 19.0 (IBM SPSS Statistics 19, SPSS Inc., Armonk, NY, USA). Mean RT and ER served as dependent variables in repeated measures 2×2×2 ANOVAs with the factors ‘task transition’ (repeat vs. switch), ‘congruence’ (congruent vs. incongruent), and ‘group’ (adolescents vs. adults). We checked for normal distribution and equal variances.

Furthermore, to examine whether there were learning or motivational effects over the course of performing the task, we analysed individuals’ cumulative ERs for the adolescent and for the adult group. We also calculated post-error slowing as RT (post-error/post-missing trials) − RT (correct trials). For this last analysis we only included participants who made at least three mistakes. This inclusion criterion was also used by Fitzgerald et al. [26].

### Functional MRI Acquisition

Data were acquired with a 3.0 T Siemens TRIO MRI scanner. For functional imaging, a standard echo planar imaging (EPI) sequence was used (repetition time (TR): 2410 ms; echo time (TE): 25 ms; flip angle: 80°). Functional MRI scans were obtained from 42 transversal slices, orientated 30° clockwise to the anterior commissure–posterior commissure line, with a thickness of 2 mm (1 mm gap), a field of view (FOV) of 192×192 mm^2^ and an in-plane resolution of 64×64 pixels, resulting in a voxel size of 3×3×3 mm^3^. Each subject had a single run with 506 TRs, leading to a duration of 20 minutes and 24 s (506 * 2410 ms). To exclude structural abnormalities, a 3D T1-weighted magnetization-prepared rapid gradient echo (MPRAGE) image data set was acquired (TR = 1900 ms, TE = 2.26 ms, FOV = 256×256 mm^2^, 176 slices, 1×1×1 mm^3^ voxel size, flip angle = 9°).

Stimuli were presented via Nordic Neurolab goggles (Bergen, Norway). The task was presented and the behavioural responses were recorded with Presentation® software (Version 11.1, Neurobehavioral Systems, Inc., Albany, CA, USA). Head motion was restricted with foam inserts that were placed to the left and the right of the head.

### Functional MRI Analysis

We analysed functional MRI data using statistical parametric mapping (SPM 5, Wellcome Department of Neuroimaging, London, United Kingdom, http://www.fil.ion.ucl.ac.uk/spm). During preprocessing, data were corrected for temporal differences in slice timing and inter-scan head motions. The scans were normalized to the standard EPI template (Montreal Neurological Institute, MNI) using a voxel size of 3×3×3 mm^3^ and smoothed with an 8 mm full-width-at-half-maximum Gaussian kernel.

First-level data analysis included the stimulus onsets of all trials as events. This within-subject procedure was used to model all effects of interest. The individual models were identical across subjects. Regressors were built from the 16 different conditions resulting from the 2 (task)×2 (task transition)×2 (previous trial congruence)×2 (present trial congruence) design. All error trials (trials in which subjects moved the joystick in the wrong direction), missing trials (trials in which subjects did not react within 2.1 seconds) as well as correct trials following error and missing trials (post-error/post-missing trials) were considered as additional regressors. To alleviate the effects of movement, we also integrated the realignment parameters (three translation and three rotation parameters) as regressors of no interest. According to the 2 (task transition)×2 (present trial congruence) design used for behavioural data analysis, all repeat and congruent trials were summed up and equally weighted irrespective of task and previous trial congruence. This was done for the other conditions analogously. Therefore, the contrasts of interest in the first level analysis were rp_C − baseline, rp_I − baseline, sw_C − baseline, sw_I − baseline, error − correct (all correct trials of the four conditions), and post-error/post-missing − correct. The resulting images were then submitted to second-level analysis.

Second-level group analysis included three different analyses. First, a full factorial whole-brain analysis including the between-subject factor group, and within-subject factors task transition and present trial congruence was calculated in order to analyse differences in task transition and congruence between adolescents and adults during correct trials. However, correct trials following error or missing trials were excluded here. This analysis mirrored the repeated measures ANOVA for the RTs and ERs (see *Behavioural data analysis*).

Second, we performed a whole-brain analysis to test whether age group affected error processing. Here we used the contrast images representing the brain response in error trials (error − correct). To test whether possible group differences with regard to error processing were mainly due to differences in performance (here ER) or not, we conducted a two-sample t-test and included the individual overall ER as a covariate. Specifically, we created one additional regressor for ER in adolescents as well as one for ER in adults, which enabled us to also model the interaction of ER and group. We only included the participants who made at least three mistakes during the whole experiment (181 adolescents and 22 adults). This inclusion criterion proved to be a reasonable trade-off between the minimum number of errors per subject to obtain a more reliable mean value over those error trials and number of participants who met the criterion. In a previous study [26] the same criterion was used.

Third, using the post-error/post-missing − correct contrast we performed a further whole-brain analysis to test whether processing of correct trials following incorrect or missing trials differed between mid-adolescents and adults. This third group statistic was analogous to the second one: We also used a two-sample t-test, included overall individual ER as a covariate with separate regressors for both groups to account for interaction, and only included participants who made at least three errors during the whole experiment.

To adjust statistical analyses to the unequal sample size we selected the ‘unequal variance’ option within SPM for all group statistics. In order to control for false positive results (type I errors) in our whole-brain analyses we used a threshold of *p*<0.05, FDR-corrected, at the voxel level and an extend threshold of at least 25 contiguous voxels. When reporting differences at this threshold we explicitly refer to significant differences. If these analyses did not yield significant group differences, we additionally ran a secondary analysis to control for false negative results (type II errors). Here we used a more lenient voxel-level threshold of *p*<0.01, uncorrected, and reported activations if they exceeded a cluster-level threshold of *p*<0.05, uncorrected. This resulted in *k* >88 voxels for the first group statistic concerning differences in task transition and present trial congruence between adolescents and adults, in *k* >67 voxels for the second group statistic concerning error processing and in *k* >75 voxels for the third group statistic concerning post-error/post-missing processing.

Behavioural as well as functional MRI data are available upon request.

### Subsample Analysis

To maximise performance differences between groups we reanalysed behavioural data comparing adults and 45 adolescents that made at least 20 mistakes during the whole task. Table 2 gives an overview of age, gender and number of errors. For the second-level fMRI group analysis we used again a full factorial design and included the between-subject factor group and the within-subject factors task transition and present trial congruence, mirroring the repeated measures ANOVA for the RTs and ERs. Again, correct trials following error or missing trials were excluded. The same thresholds for fMRI analysis were used resulting in *k* >79 voxels for the lenient threshold.

**Table 2:** Details of the adolescent subsample (adolescents that made at least 20 mistakes) and the adult sample.

		Adolescent subsample	Adults
Age	Range	14.0–15.0	20–50
	Mean [SD]	14.6 [0.3]	25.1 [6.4]
Gender	Male	22	16
	Female	23	12
Subsample size		45	28
Errors	Range	20–50	0–21
	Mean [SD]	27.9 [6.6]	5.9 [5.0]

## Results

### Behavioural Data

**Figure 2:**
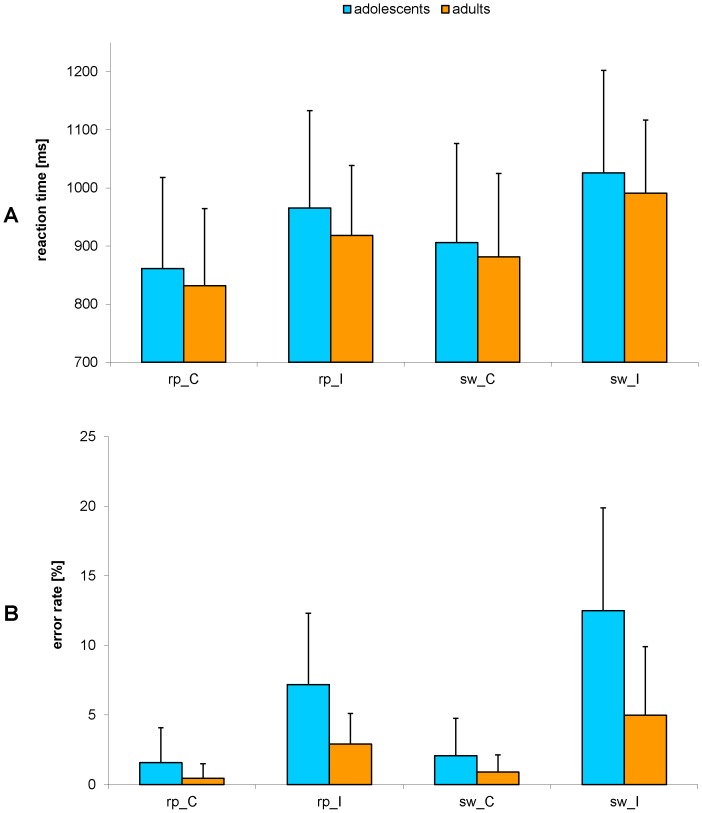
Mean reaction times (A) and error rates (B) for the four different conditions resulting from the factors task transition and congruence in adolescents (blue) and adults (orange). Notes: rp – repeat, sw – switch, C – congruent, I – incongruent. The error bars indicate the area of one standard deviation around the mean.

The repeated measures ANOVA was calculated in order to analyse the effects of task transition and congruence in adolescents and adults. Mean RTs and ERs for adults and adolescents for the four different conditions are depicted in Figure 2.

Subjects reacted slower in switch (*F*(1,211) = 230.503; *p*<0.001; η_p_^2^ = 0.522) and incongruent trials (*F*(1,211) = 279.488; *p*<0.001; η_p_^2^ = 0.570), compared to repeat and congruent trials, respectively. Additionally, there was a significant interaction of task transition * congruence (*F*(1,211) = 15.711; *p*<0.001; η_p_^2^ = 0.069), indicating that the effect of incongruence was enhanced in switch compared to repeat trials. There were no significant differences in RT between adolescents and adults (*F*(1,211) = 0.976; *p* = 0.324; η_p_^2^ = 0.005) as well as no significant interaction of group and task transition (*F*(1,211) = 0.261; *p* = 0.610; η_p_^2^ = 0.001), of group and congruence (*F*(1,211) = 0.596; *p* = 0.441; η_p_^2^ = 0.003), and of all three factors (*F*(1,211) = 0.047; *p* = 0.829; η_p_^2^<0.001).

In contrast to mean RT, ER showed a significant main effect of group (*F*(1,211) = 26.715; *p*<0.001; η_p_^2^ = 0.112) with adolescents making significantly more mistakes than adults (overall ER for adolescents: 5.8%, for adults: 2.3%). The effect of task transition as well as the effect of congruence were enhanced in adolescents compared to adults (*F*(1,211) = 6.040; *p* = 0.015; η_p_^2^ = 0.028; Δ_rp_ = 2.7%; Δ_sw_ = 4.4%; and *F*(1,211) = 24.065; *p*<0.001; η_p_^2^ = 0.102; Δ_C_ = 1.1%; Δ_I_ = 5.9%). Further, there was a significant three-way interaction of group, task transition and congruence (*F*(1,211) = 8.147; *p* = 0.005; η_p_^2^ = 0.037), meaning that the interaction between task transition and congruence is enhanced in adolescents compared to adults. Subjects were less accurate in switch trials (*F*(1,211) = 38.899; *p*<0.001; η_p_^2^ = 0.156) and in incongruent trials (*F*(1,211) = 135.944; *p*<0.001; η_p_^2^ = 0.392). Again, the effect of congruence was larger in switch than in repeat trials (*F*(1,211) = 33.183; *p*<0.001; η_p_^2^ = 0.136).

Since adolescents made more mistakes than adults, we examined the course of the cumulative mean error during the interference and switch task. The error frequency remained constant over the task, indicated by a nearly perfect linear growth pattern of errors for both groups (R^2^>0.99).

Further, we observed a significant post-error slowing effect (*Z* = −6.821, *p*<0.001; mean PES: 43.59 ms; N = 181 adolescents and N = 22 adults), but no significant differences between adolescents and adults (*Z* = −0.158, *p* = 0.875). There was no correlation between ER and post-error slowing (ρ = −0.027, p = 0.669).

Results did not change when reanalysing the data with a subsample of 45 adolescents with at least 20 errors.

In summary, adolescents reacted as fast as adults and showed equal post-error slowing effects, but were more susceptible to task transition and congruence effects in terms of mistakes.

### Brain Response during Correct Trials

Concerning the main effect of group brain activity during correct trials did not yield any clusters with stronger brain responses in adolescents than in adults. Even when using a very liberal threshold (*p*<0.01, uncorrected, at voxel-level, and *p*<0.05, uncorrected, at cluster-level, i.e. *k* >88 voxels) to reduce the type II errors, no differences in this direction could be detected. On the other hand adolescents showed a significantly weaker brain response than adults in the right cerebellum.

**Figure 3:**
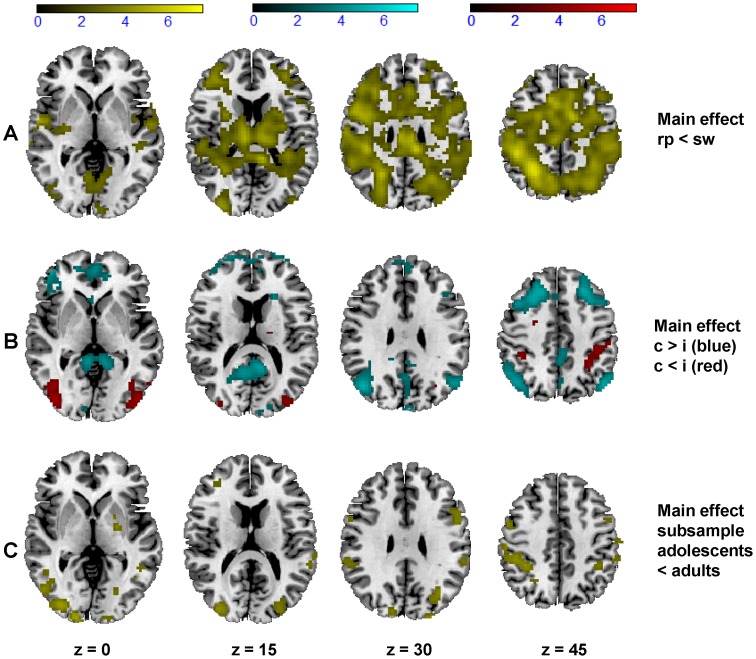
Brain response during correct trials. A) Main effect of task transition: Regions of the brain that respond more strongly during switch compared to repeat trials in adolescents and adults (threshold T = 2.11, p<0.05, FDR-corrected, in 25 contiguous voxels, yellow colour scale). B) Main effect of congruence: Regions of the brain respond more strongly during congruent compared to incongruent trials in adolescents and adults (threshold T = 2.64, p<0.05, FDR-corrected, in 25 contiguous voxels, blue colour scale), and regions of the brain that respond more strongly during incongruent compared to congruent trials in adolescents and adults (threshold T = 2.97, p<0.05, FDR-corrected, in 25 contiguous voxels, red colour scale). C) Main effect of group in the subsample analysis (N = 45 error-prone adolescents and N = 28 adults): Regions of the brain that respond weaker in adolescents compared to adults (threshold T = 2.87, p<0.05, FDR-corrected, in 25 contiguous voxels, yellow colour scale).

Regarding the main effect of task transition brain responses during switch compared to repeat trials were significantly enhanced in a well-known network of parietal and prefrontal structures (Figure 3A, for details see Table S1). No significant differences were found in the opposite direction, even at a lenient threshold. Further, there was a main effect of congruence: Incongruent in contrast to congruent trials revealed significantly stronger brain responses in bilateral occipital regions, in the right MFG (BA 6), in the bilateral superior parietal lobe (BA 7) and in the left inferior parietal lobe (BA 40). Contrariwise, brain responses during incongruent trials were weaker in several frontal, occipital, temporal and parietal regions (Figure 3B, for further details see Table S1).

There were no significant interactions between group and task transition, however at a lenient threshold, analyses revealed enhanced neural switch costs in adolescents compared to adults in several brain regions including e.g. right ACC, and right MFG (BA 6). No significant interactions between group and congruence or between all three factors were observed even when applying a liberal threshold. However, there was a significant interaction between task switch and incongruence in a cluster within the cerebellum and midbrain, in a frontal cluster including the right ACC (BA 24), in a cluster including the left parahippocampal gyrus, in the thalamus, in the cingulate gyrus, and in a small cluster including the right parahippocampal gyrus (BA 20).

Reanalysing the subsample of adolescents (N = 45 adolescents that made at least 20 mistakes and N = 28 adults) yielded changes within the group main effect and within the interaction between group and task transition, and group and congruence respectively (see Table S2). Error-prone adolescents showed a significant weaker brain response in the pre-SMA (BA 6), in parietal regions (BA 7, BA 40), in the MFG (BA 45), in several occipital regions and in the cerebellum (see Figure 3C), but no differences in the opposite direction. Again, interactions with group were only observed at a liberal threshold: Neural switch costs were enhanced in error-prone adolescents in the left occipital gyrus (BA 19), and neural incongruence effects were enhanced in adolescents in the right inferior frontal gyrus (BA 47). There was also an interaction of all three factors in a way that adults showed a higher brain response than error-prone adolescents in the cingulate cortex when neural switch costs and incongruence effects co-occurred.

### Brain Response during Error Trials

Note that only data from the 181 adolescents and 22 adults who made at least three mistakes were included in the two group statistics concerning error trials and post-error/post-missing trials (see *Materials and methods*). ER was included as a covariate in both statistics with two regressors to account for interaction effects. Regarding brain response during error trials, the analysis revealed a well-known network consisting of parietal and frontal cortices (including insula, PPC, and pre-SMA; for further details see Table S3), but only at an uncorrected threshold (*p*<0.01, voxel-level, uncorrected, and *p*<0.05, cluster-level, uncorrected, i.e. *k* >67 voxels). When controlled for ER, brain response in adolescents and adults during error processing did not differ significantly, even at a liberal threshold.

**Figure 4:**
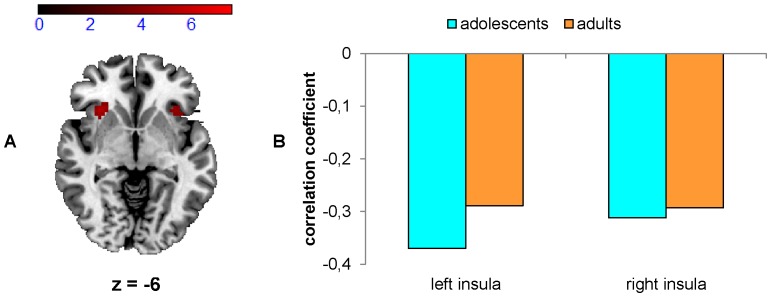
Brain response during error trials. Note that only 181 adolescents and 22 adults who made at least 3 mistakes were considered for this analysis. A) Regions of the brain during error trials that show a significant negative correlation with overall ER (threshold T = 3.84, p<0.05, FDR-corrected, in 25 contiguous voxels) in adolescents. B) Correlation coefficients for the negative correlation between brain response during error trials and overall ER for adolescents (blue) and adults (orange) in the peak voxels (please see also Table S3). The correlation only reached significance in adolescents.

Notably, there was a significant negative correlation between overall ER and brain response during error trials within bilateral anterior insulae for adolescents (see Figure 4A, red, and Table S3), although a trend in the same direction can also be seen for adults (see Figure 4B). There were no significant positive correlations between ER and brain responses during error trials, and no significant interaction between group and ER.

Applying an uncorrected threshold only revealed a positive correlation in adolescents between ER and brain response during error trials within the right cuneus, the left supramarginal gyrus, the right paracentral lobule (BA 4) and the left cerebellum.

### Brain Response during Correct Post-error/Post-missing Trials

Regarding brain responses during correct trials following incorrect or missing responses (compared to other correct trials), our analysis revealed a significantly stronger brain response in a network of frontal and parietal areas (see Table S4). When controlled for performance in terms of ER, brain response in both groups during post-error/post-missing processing did not differ significantly. At a less conservative threshold (*p*<0.01, uncorrected, at voxel-level, and *p*<0.05, uncorrected, at cluster-level, i.e. *k* >75 voxels) this analysis revealed a weaker brain response in adolescents in the left MFG (BA 10) (see Table S4).

**Figure 5:**
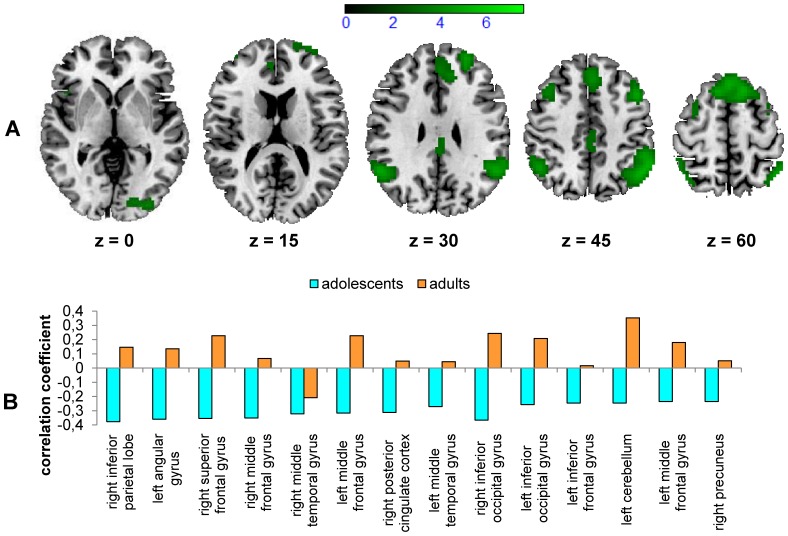
Brain response during correct post-error/post-missing trials. Note that only 181 adolescents and 22 adults which made at least three mistakes were considered for this analysis. A) Regions of the brain during post-error/post-missing trials that show a significant negative correlation with overall ER (threshold T = 2.77, p<0.05, FDR-corrected, in 25 contiguous voxels) in adolescents. B) Correlation coefficients for the correlation between brain response during post-error/post-missing trials and overall ER for adolescents (blue) and adults (orange) in the peak voxels (sorted by t-values, please see also Table S4). The correlations only reached significance in adolescents.

We find it interesting that there was a significant negative correlation between brain response during post-error/post-missing trials and overall ER in a network of parietal and prefrontal structures (see Figure 5A, green colour scale, and Table S4), but only in adolescents. For adults there was a non-significant trend in the opposite direction (see Figure 5B). Although not significant at the predefined level, the secondary analysis at a lenient threshold revealed an interaction between ER and group in the left superior frontal gyrus (BA 6).

## Discussion

As expected, adolescents in general made more errors than adults in the combined interference and switch task and exhibited stronger increases in ER in trials with higher cognitive demands, i.e. in switch and incongruent trials. Nevertheless, during correct trials RTs did not differ and brain responses were widely comparable for both groups and only less pronounced in the right cerebellum of adolescents compared to adults. When analysing a subsample of 45 adolescents with more than 20 errors (i.e. a 4.7-fold higher ER) brain response in adolescents in the pre-SMA and in the PPC was weaker than in adults.

Regarding the neural correlate of a 2.5-fold higher ER in mid-adolescents we found that within this group ER was negatively correlated with brain response in the left and right insulae during error trials and in a network of parietal and prefrontal areas during those trials following errors or missing responses. When controlled for performance differences (ER) during error and post-error/post-missing trials brain responses did not differ between groups. We conclude that adolescents with a stronger brain response in these trials are better at monitoring their performance and after detecting errors are more capable of flexibly allocating additional cognitive control resources, and thus they make fewer errors than their peers.

### Behavioural and Neural Effects of Task Transition and Congruence in General

As we expected, RT and ER substantially increased in switch compared to repeat trials. Likewise, RT and ER were higher in incongruent compared to congruent trials, and this increase was particularly pronounced on switch trials. This is in agreement with previous studies [5], [7], [11], [13] and indicates that our task is suitable for examining cognitive control.

In line with these behavioural parameters, in the fMRI data we found substantially stronger BOLD response during switch compared to repeat trials in a well-described brain network including frontal and parietal cortices [5], [17], [19]. A result that we had not expected was that for congruent compared to incongruent trials activation was higher in several frontal and parietal regions, namely the posterior cingulate gyrus, medial frontal regions, and occipital regions. These brain areas have been associated with the so-called default-mode network and are less activated during demanding cognitive tasks [47]. As no conflict should be experienced during congruent trials, we therefore speculate that brain response within this default-mode network increases [48]. Further, incongruent in contrast to congruent trials showed activation increase in the bilateral PPC (BA 7, BA 40) as well as in the right MFG and in the left superior frontal gyrus (both BA 6), which is in line with results from prior studies [16], [18], [19]. However, no brain response in the ACC or dlPFC was found, which contradicts previous findings [9], [12], [14]. As this study focused on differences between adults and adolescents in cognitive control, the issue of brain response in the ACC and dlPFC will be further addressed in future investigations (Mennigen et al., in preparation).

In summary, the task successfully produced effects of task transition and congruence, as shown by behavioural and imaging data.

### Behavioural Differences between Adolescents and Adults

Although adolescents and adults reacted equally fast, adolescents’ ER was 2.5 times larger than the adults’ ER. This is consistent with prior studies [27], [31], [37], [49]. ERs were stable over the time course of the experiment in adolescents as well as in adults, indicating that differences in ER cannot be explained by differential exhaustion effects.

### Comparable Brain Response during Correct Trials

We were surprised to find that neural activation during correct trials only revealed a weaker brain response within the right cerebellum for adolescents. But, when comparing error-prone adolescents to adults the subsample analysis revealed additionally a significant weaker brain response within the pre-SMA, the PPC, the MFG, and in occipital regions in the younger group. Prior studies [5], [50], [51] also reported cerebellar activity during cognitive control tasks leading to the speculation that the cerebellum also plays a “crucial role in conflict processing” [52], [53]. So, one could speculate that the adolescents’ lower engagement of the cerebellum during correct trials may result in a higher susceptibility to commit errors.

When we applied a liberal threshold (*p*<0.01, voxel-level, uncorrected, and *p*<0.05, cluster-level, uncorrected, i.e. *k* >88 voxels) we found that neural switch costs were enhanced in adolescents compared to adults in occipital and temporal regions, in the right ACC, and in the right MFG. Within the subsample analysis (N = 45 error-prone adolescents and N = 28 adults) only the differences in the occipital regions remained and additionally the neural incongruence effect was enhanced in error-prone adolescents in the right inferior frontal gyrus. Thus, we speculate that during highly demanding switch or incongruent trials adolescents’ brain activation might increase in parts of the cognitive control network resulting in the same performance for both groups. However, these differences have to be interpreted with caution as they were based on an analysis in which we applied a liberal threshold to reduce the risk of type II errors.

In line with previous findings [27], [28], [31], we obtained significant group differences in expected frontal and parietal regions such as the dlPFC, pre-SMA or PPC, but only when maximising performance differences between groups. Taking into account the whole adolescent sample for data analysis, group differences in regions of the cognitive control network vanished. A reason for the discrepant results may be that similar but not identical tasks were used and that prior studies investigated a broader age range, resulting in greater performance differences between groups. Further, differences were found via regression analyses. As we aimed at examining a more age-homogenous group of adolescents, regression analyses were not feasible, because they require a normal distribution of the variables [54]. In light of structural brain development [22], [24] it seems plausible that there is a positive correlation between age and brain activation resulting in a weaker brain response in adolescents than in adults which is paralleled by a worse performance of the younger group.

Assuming that our task is well suited to examining cognitive control, we conclude from our present findings that adolescents’ maturity of the cognitive control network is performance-dependent as developmental differences were only found in the subgroup of error-prone adolescents. This leads to the speculation that there is a great interindividual variance in the development of cognitive control. We further conclude that, when taking into account results of the whole adolescent sample, by the age of 14 the majority of adolescents recruit the same brain regions as adults when performing the interference and switch task correctly. Future investigations should thus examine more balanced samples and preferentially conduct longitudinal analyses to uncover neural correlates of the adolescent development of cognitive control.

### Brain Responses during Error Trials

Independent of group, we found stronger brain responses during error trials (compared to correct trials) in parietal cortices, the insula, and the dorsal ACC. Although only evident at a liberal threshold, the error processing activity is in line with previous studies [15], [32], [35], [55]–[57]. One reason for not finding a more pronounced activity during error processing may be the chosen statistical design, i.e., that we controlled for performance (ER) in this analysis and some of the error processing variance is explained by this covariate.

Unlike some prior developmental studies [26], [35]–[37], we did not find any (significant) differences in neural correlates of error processing between adults and adolescents. But, our results are in line with other previous studies [35], [58], which also found no activation differences within the ACC between different age groups.

What is more, we obtained a significant negative correlation in adolescents between brain response in the bilateral anterior insulae during error processing and ER, i.e., the higher the insula activation during error trials, the lower the ER. For adults, there was only a trend, which may be due to their smaller variance of ER and of error-related activity, and to their substantially smaller sample size. At a liberal threshold there was also a correlation in the positive direction for adolescents in the right cuneus, the left supramarginal gyrus, the right paracentral lobule (BA 4) and the left cerebellum. To our knowledge only three studies correlated activity during error trials with ER: Fitzgerald et al. [26] revealed a negative correlation between ER and brain response in the dorsal ACC. Abel et al. [59] did not find any correlation, whereas Hester et al. [32] found higher activation in the right insula within the subgroup that committed most errors. However, the latter peak activation is located more posterior (MNI peak coordinates: 40/−13/−3) than our activation (MNI peak coordinates: −33/21/−6 and 33/24/0). In light of previous evidence suggesting that the insula is part of the error detection network [32], [35], [55], we conclude that adolescents’ inter-individual differences in ER may partly be explained by insular activation. As the insula is further associated with error awareness [60], we speculate that adolescents with higher insula activation were more aware of their errors, resulting in a lower ER. Unfortunately, we did not ask participants after each trial whether they felt that they had committed an error or not. This should be done in future investigations. The association of error-induced insula activation and ER in adolescents indicates that neural error processing might be particularly immature in adolescents with poor performance. But, this claim can only be addressed in our planned longitudinal analyses.

### Brain Response during Post-error/Post-missing Trials

Behaviourally, we found a significant post-error slowing effect [34], [45], [46]. Previous studies suggest that this effect reflects an orienting response requiring (cognitive) control mechanisms [34] which is evident in increased neural activity in frontal and parietal areas. In line with that, we found a significantly stronger brain response during correct trials following incorrect or missing responses (compared to other correct trials) in a well-known network of frontal and parietal regions, i.e. in the so-called cognitive control network [21].

However, there were no significant neural or behavioural differences between adolescents and adults, although a prior study [26] reported a lower left inferior frontal gyrus activation in adolescents compared to adults during post-error trials. As the cognitive control network was activated independent of group, and differences between adolescents and adults were only found in the left MFG at a liberal threshold, we conclude that both adolescents and adults show post-error adjustment. Future studies have to recruit more balanced samples and use a more challenging task producing higher ERs.

It is important to note that only for adolescents was there a significant negative correlation between overall ER and brain response during post-error/post-missing trials in several regions of the cognitive control network, e.g. the bilateral inferior parietal lobe (BA 39), the bilateral MFG (BA 9, BA 46), the right superior frontal gyrus (BA 8) and the posterior cingulate cortex (BA 23). For adults there was a trend in the opposite direction, which is further indicated by the interaction between ER and group at the liberal threshold in the left superior frontal gyrus (BA 6). To our knowledge, no study has reported a correlation between post-error brain response and ER. Indeed, King et al. [34] also analysed neural post-error effects in adults, but they entered behavioural post-error slowing as well as post-error reduction of interference as covariates, which are both RT measures. They reported a positive correlation in the right inferior frontal junction as well as a negative correlation in the sensorimotor cortex and in the fusiform face area for post-error slowing, and a positive correlation in the superior frontal sulcus and the fusiform face area for post-error reduction of interference. Fitzgerald et al. [26] did not find any correlation between ER and brain response during post-error trials. Thus, we speculate that the correlation between neural post-error adjustment and behavioural ER in adolescents is due to individual developmental differences in this age group: Adolescents that respond more strongly in correct post-error/post-missing trials within the above-mentioned brain areas may be more able to adjust their cognitive control, resulting in a lower overall ER, meaning a better task performance. Again, the question if this implicates that the cognitive control network in adolescents with poor performance is not yet fully mature will be addressed in our longitudinal analysis.

### Limitations

Several limitations of the present study should be mentioned. First, we used a cross-sectional study design. These designs are confounded by inter-individual cohort and variance effects, which can weaken true developmental effects [22]. However, as our study is planned as a longitudinal design, adolescents will be investigated again at the age of 16 and 18. Second, although we examined an unusually large sample of adolescents, the small adult sample limits the power of our results. Moreover, our adolescent sample is not representative regarding level of education. In our sample, 69% of the adolescents attended a ‘Gymnasium’, a school which has selective entry requirements based on academic ability. The remaining 31% attended a ‘Mittelschule’, for pupils of medium academic ability. This ratio is in contrast to that of the state of Saxony, in which approximately 50% of adolescents attend a ‘Gymnasium’, and results from those pupils being more willing to participate in the study than the pupils from the ‘Mittelschule’. Further, our adult sample is highly educated (85% of the adults are university students), which might affect the magnitude of differences between adults and adolescents. Unfortunately, we did not collect any data to match groups for further covariates, such as intelligence or socioeconomic status. This should be done in future investigations.

## Conclusions

Although both groups reacted equally fast during correct trials of our interference and switch task, adolescents made 2.5 times as many mistakes as adults. In line with their similar performance during correct trials, the majority of adolescents and adults engage their cognitive control network to the same extent.

Regarding error processing, two mechanisms seem to be pivotal: First, error monitoring and second, the post-error adjustments during trials directly following errors. We found it interesting that during error trials overall ER correlated negatively with brain response in the anterior insulae, and during post-error/post-missing trials overall ER correlated negatively with brain response in several regions of the cognitive control network, but only for adolescents. We conclude that adolescents that commit fewer errors than their peers might be better at monitoring their performance, indicated by a stronger brain response during error trials, and might also subsequently be more capable of flexibly allocating additional cognitive control resources, as mirrored by the more pronounced activity of their cognitive control network after incorrect or missing responses. One could further speculate that, especially in adolescents making many mistakes, neural (post-)error processing is still less mature.

## Supporting Information

Table S1 **Increase in brain response for the first group statistic concerning task effects during correct trials (N = 185 adolescents and N = 28 adults).** If there were no significant differences at the corrected threshold, we additionally report results from the exploratory analysis (p<0.01, uncorrected, voxel-level, and p<0.05, uncorrected, cluster-level, i.e. *k* >88 voxels). The following abbreviations are used: repeat (rp), switch (sw), congruent trial (C), incongruent trial (I).
(DOCX)

Table S2 **Increase in brain response for the group statistic concerning task effects during correct trials (subsample analysis, N = 45 adolescents that made at least 20 mistakes and N = 28 adults).** If there were no significant differences at the corrected threshold, we additionally report results from the exploratory analysis (p<0.01, uncorrected, voxel-level, and p<0.05, uncorrected, cluster-level, i.e. *k* >79 voxels). The following abbreviations are used: repeat (rp), switch (sw), congruent trial (C), incongruent trial (I).
(DOCX)

Table S3 **Increase in brain response for the second group statistic concerning error trials (N = 181 adolescents and N = 22 adults).** If there were no significant differences at the corrected threshold, we additionally report results from the exploratory analysis (p<0.01, uncorrected, voxel-level, and p<0.05, uncorrected, cluster-level, i.e. *k* >67 voxels). The following abbreviations are used: overall error rate (ER).
(DOCX)

Table S4 **Increase in brain response for the third group statistic concerning correct trials following errors or missing trials (N = 181 adolescents and N = 22 adults).** If there were no significant differences at the corrected threshold, we additionally report results from the exploratory analysis (p<0.01, uncorrected, voxel-level, and p<0.05, uncorrected, cluster-level, i.e. *k* >75 voxels). The following abbreviations are used: overall error rate (ER), supplementary motor area (SMA).
(DOCX)
