# Supplementary material for: Interindividual Differences in Mid-Adolescents in Error Monitoring and Post-Error Adjustment
Source: PLoS One. 2014 Feb 18;9(2):e88957. doi: 10.1371/journal.pone.0088957 (PMC3928333; doi:10.1371/journal.pone.0088957)
Supplement: Table S1 — Increase in brain response for the first group statistic concerning task effects during correct trials (N = 185 adolescents and N = 28 adults). If there were no significant differences at the corrected threshold, we additionally report results from the exploratory analysis (p<0.01, uncorrected, voxel-level, and p<0.05, uncorrected, cluster-level, i.e. k >88 voxels). The following abbreviations are used: repeat (rp), switch (sw), congruent trial (C), incongruent trial (I). (DOCX) [file pone.0088957.s001.docx]

Supplementary Table S1. Increase in brain response for the first group statistic concerning task effects during correct trials (N=185 adolescents and N=28 adults).

| Contrast | p value (voxel-level) | Primary peak location | Hemisphere | Brodmann’s areas | MNI coordinates | | | t | Cluster p (cor.) | Cluster p (unc.) | Cluster size (voxels) |
| --- | --- | --- | --- | --- | --- | --- | --- | --- | --- | --- | --- |
|  |  |  |  |  | x | y | z |  |  |  |  |
| adolescents > adults | fdr 0.05, unc. 0.01 | no suprathreshold clusters | | | | | | | | | |
| adolescents < adults | fdr 0.05 | Cerebellum | right |  | 36 | -57 | -21 | 5.34 | 0.008 | 0.007 | 44 |
| rp > sw | fdr 0.05, unc. 0.01 | no suprathreshold clusters | | | | | | | | | |
| rp < sw | fdr 0.05 | Frontoparietal cluster: |  |  |  |  |  |  | 0.000 | 0.000 | 18817 |
|  |  | - Inferior parietal lobe | left | BA 7 | -27 | -57 | 39 | 7.36 |  |  |  |
|  |  | - Superior frontal gyrus | left | BA 6 | -24 | -3 | 54 | 6.27 |  |  |  |
|  |  | - Inferior parietal lobe | left | BA 40 | -45 | -39 | 45 | 6.22 |  |  |  |
|  |  | Fusiform gyrus | left |  | -30 | -3 | -33 | 3.25 | 1.000 | 0.352 | 25 |
|  |  | Temporal lobe, sub-gyral | left |  | -42 | -15 | -18 | 3.16 | 1.000 | 0.234 | 41 |
|  |  | Cerebellum | left |  | -12 | -81 | -27 | 3.02 | 1.000 | 0.284 | 33 |
|  |  | Cerebellum | right |  | 12 | -78 | -24 | 2.94 | 1.000 | 0.251 | 38 |
| C > I | fdr 0.05 | Superior frontal gyrus | left | BA 8 | -18 | 27 | 51 | 6.49 | 0.000 | 0.000 | 722 |
|  |  | Superior frontal gyrus | right | BA 8 | 24 | 30 | 51 | 5.97 | 0.000 | 0.000 | 429 |
|  |  | Angular gyrus | left | BA 39 | -45 | -72 | 33 | 5.64 | 0.000 | 0.000 | 453 |
|  |  | Hippocampus | left | BA 20 | -24 | -18 | -18 | 5.43 | 0.020 | 0.001 | 203 |
|  |  | Angular gyrus | right | BA 39 | 45 | -69 | 42 | 5.36 | 0.021 | 0.001 | 201 |
|  |  | Posterior cingulate gyrus | left | BA 29 | -9 | -48 | 6 | 5.13 | 0.000 | 0.000 | 954 |
|  |  | Middle temporal gyrus | left | BA 20 | -45 | 6 | -27 | 4.94 | 0.144 | 0.008 | 119 |
|  |  | Parahippocampal gyrus | right | BA 35 | 18 | -9 | -24 | 4.83 | 0.010 | 0.001 | 234 |
|  |  | Medial frontal gyrus | right | BA 11 | 6 | 24 | -9 | 4.71 | 0.000 | 0.000 | 858 |
|  |  | Inferior frontal gyrus | right | BA 47 | 36 | 33 | -12 | 4.24 | 0.851 | 0.103 | 39 |
|  |  | Superior frontal gyrus | right | BA 10 | 21 | 66 | 9 | 4.22 | 0.680 | 0.062 | 53 |
|  |  | Occipital lobe | right |  | 3 | -90 | 33 | 3.83 | 0.641 | 0.056 | 56 |
|  |  | Inferior temporal gyrus | right | BA 20 | 57 | -15 | -24 | 3.70 | 0.756 | 0.076 | 47 |
|  |  | Frontal gyrus, sub-lobar | right |  | 21 | 21 | 15 | 3.67 | 0.817 | 0.092 | 42 |
|  |  | Middle temporal gyrus | right | BA 20 | 48 | 3 | -24 | 3.51 | 0.962 | 0.177 | 26 |
|  |  | Lingual gyrus | left | BA 18 | -12 | -81 | -6 | 3.20 | 0.829 | 0.096 | 41 |
| C < I | fdr 0.05 | Inferior occipital gyrus | left | BA 19 | -42 | -72 | -9 | 5.34 | 0.000 | 0.000 | 261 |
|  |  | Inferior occipital gyrus | right | BA 19 | 45 | -75 | -9 | 5.20 | 0.000 | 0.000 | 429 |
|  |  | Middle frontal gyrus | right | BA 6 | 30 | -6 | 54 | 4.61 | 0.022 | 0.002 | 115 |
|  |  | Cerebellum | right |  | 33 | -51 | -27 | 4.61 | 0.066 | 0.007 | 85 |
|  |  | Superior frontal gyrus | left | BA 6 | -24 | -9 | 60 | 4.37 | 0.003 | 0.000 | 173 |
|  |  | Thalamus | right |  | 15 | -15 | 12 | 4.36 | 0.628 | 0.108 | 26 |
|  |  | Superior parietal lobe | left | BA 7 | -18 | -63 | 57 | 4.34 | 0.006 | 0.001 | 156 |
|  |  | Superior parietal lobe | right | BA 7 | 21 | -66 | 57 | 4.30 | 0.001 | 0.000 | 237 |
|  |  | Inferior parietal lobe | left | BA 40 | -39 | -39 | 45 | 3.98 | 0.472 | 0.070 | 34 |
| adolescents > adults (sw − rp) | fdr 0.05 | no suprathreshold clusters | | | | | | | | | |
|  | unc. 0.01 | Inferior temporal lobe | left | BA 20 | -45 | -24 | -21 | 4.87 | 0.616 | 0.031 | 109 |
|  |  | Superior temporal gyrus | right | BA 48 | 60 | -9 | 3 | 4.69 | 0.007 | 0.000 | 406 |
|  |  | Superior temporal gyrus | left | BA 48 | -60 | -6 | 0 | 4.50 | 0.272 | 165 | 165 |
|  |  | Occipital cluster: |  |  |  |  |  |  | 0.000 | 0.000 | 1442 |
|  |  | - Cuneus | right | BA 19 | 15 | -84 | 24 | 4.10 |  |  |  |
|  |  | - Lingual gyrus | right | BA 17 | 9 | -66 | 6 | 3.89 |  |  |  |
|  |  | - Inferior occipital gyrus | left | BA 19 | -45 | -69 | -15 | 3.85 |  |  |  |
|  |  | Cingulate gyrus | right |  | 9 | -15 | 48 | 3.78 | 0.021 | 0.001 | 330 |
|  |  | Anterior cingulate gyrus | right |  | 6 | 21 | 21 | 3.52 | 0.290 | 0.011 | 161 |
|  |  | Middle frontal gyrus | right | BA 6 | 42 | -3 | 60 | 3.13 | 0.518 | 0.024 | 122 |
| adolescents < adults (sw − rp) | fdr 0.05, unc. 0.01 | no suprathreshold clusters | | | | | | | | | |
| adolescents > adults (I − C) | fdr 0.05, unc. 0.01 | no suprathreshold clusters | | | | | | | | | |
| adolescents < adults (I − C) | fdr 0.05, unc. 0.01 | no suprathreshold clusters | | | | | | | | | |
| interaction sw>rp & I> C | fdr 0.05 | Lingual gyrus | right | BA 27 | 6 | -39 | 0 | 5.02 | 0.000 | 0.000 | 411 |
|  |  | Frontal lobe, sub-gyral | right |  | 21 | 36 | 12 | 4.46 | 0.001 | 0.000 | 205 |
|  |  | Sub-lobar, extra-nuclear | left |  | -21 | -15 | -9 | 4.46 | 0.000 | 0.000 | 260 |
|  |  | Thalamus | left/right |  | 0 | -9 | 6 | 4.37 | 0.431 | 0.077 | 29 |
|  |  | Cingulate gyrus | left/right |  | 0 | -15 | 30 | 4.31 | 0.080 | 0.011 | 67 |
|  |  | Parahippocampal gyrus | right | BA 20 | 33 | -18 | -24 | 3.79 | 0.378 | 0.065 | 32 |
| adolescents > adults (interaction sw>rp & I>C) | fdr 0.05, unc. 0.01 | no suprathreshold clusters | | | | | | | | | |
| adolescents < adults (interaction sw>rp & I>C) | fdr 0.05, unc. 0.01 | no suprathreshold clusters | | | | | | | | | |

If there were no significant differences at the corrected threshold, we additionally report results from the exploratory analysis (p < 0.01, uncorrected, voxel-level, and p < 0.05, uncorrected, cluster-level, i.e. *k* > 88 voxels). The following abbreviations are used: repeat (rp), switch (sw), congruent trial (C), incongruent trial (I).
