# Supplementary material for: Interindividual Differences in Mid-Adolescents in Error Monitoring and Post-Error Adjustment
Source: PLoS One. 2014 Feb 18;9(2):e88957. doi: 10.1371/journal.pone.0088957 (PMC3928333; doi:10.1371/journal.pone.0088957)
Supplement: Table S2 — Increase in brain response for the group statistic concerning task effects during correct trials (subsample analysis, N = 45 adolescents that made at least 20 mistakes and N = 28 adults). If there were no significant differences at the corrected threshold, we additionally report results from the exploratory analysis (p<0.01, uncorrected, voxel-level, and p<0.05, uncorrected, cluster-level, i.e. k >79 voxels). The following abbreviations are used: repeat (rp), switch (sw), congruent trial (C), incongruent trial (I). (DOCX) [file pone.0088957.s002.docx]

Supplementary Table S2. Increase in brain response for the group statistic concerning task effects during correct trials (subsample analysis, N=45 adolescents that made at least 20 mistakes and N=28 adults).

| Contrast | p value (voxel-level) | Primary peak location | Hemisphere | Brodmann’s areas | MNI coordinates | | | t | Cluster p (cor.) | Cluster p (unc.) | Cluster size (voxels) |
| --- | --- | --- | --- | --- | --- | --- | --- | --- | --- | --- | --- |
|  |  |  |  |  | x | y | z |  |  |  |  |
| adolescents > adults | fdr 0.05, unc. 0.01 | no suprathreshold clusters | | | | | | | | | |
| adolescents < adults | fdr 0.05 | Fusiform gyrus/Cerebellum | right | BA 37 | 39 | -57 | -21 | 6.01 | 0.000 | 0.000 | 444 |
|  |  | Precentral gyrus | right | BA 6 | 51 | 3 | 36 | 4.98 | 0.010 | 0.001 | 148 |
|  |  | Primary visual cortex | left | BA 17 | -18 | -99 | -3 | 4.71 | 0.003 | 0.000 | 187 |
|  |  | Inferior parietal lobe | left | BA 40 | -45 | -39 | 51 | 4.60 | 0.000 | 0.000 | 441 |
|  |  | Middle occipital gyrus | left | BA 19 | -33 | -87 | 0 | 4.47 | 0.000 | 0.000 | 401 |
|  |  | Superior occipital gyrus | right | BA 18 | 24 | -93 | 27 | 4.16 | 0.004 | 0.000 | 180 |
|  |  | Putamen | right |  | 24 | -6 | 3 | 4.14 | 0.651 | 0.075 | 32 |
|  |  | Superior parietal lobe | right | BA 7 | 24 | -63 | 57 | 4.11 | 0.064 | 0.005 | 95 |
|  |  | Superior temporal gyrus | right | BA 42 | 63 | -33 | 21 | 4.04 | 0.002 | 0.000 | 208 |
|  |  | Precentral gyrus | left | BA 6 | -57 | 3 | 39 | 3.92 | 0.077 | 0.006 | 90 |
|  |  | Postcentral gyrus | right | BA 3 | 57 | -15 | 39 | 3.87 | 0.751 | 0.099 | 27 |
|  |  | Middle frontal gyrus | left | BA 45 | -42 | 39 | 21 | 3.70 | 0.419 | 0.039 | 45 |
| rp > sw | fdr 0.05, unc. 0.01 | no suprathreshold clusters | | | | | | | | | |
| rp < sw | fdr 0.05 | Frontoparietal cluster: |  |  |  |  |  |  | 0.000 | 0.000 | 6425 |
|  |  | - Superior frontal gyrus | left | BA 6 | -24 | -3 | 54 | 5.43 |  |  |  |
|  |  | - Inferior parietal lobe | left | BA 40 | -45 | -39 | 42 | 5.29 |  |  |  |
|  |  | - Inferior parietal lobe | left | BA 7 | -27 | -57 | 39 | 5.27 |  |  |  |
|  |  | Precentral gyrus | right | BA 44 | 45 | 3 | 30 | 4.15 | 0.031 | 0.001 | 212 |
|  |  | Precentral gyrus | left | BA 44 | -48 | 3 | 30 | 4.07 | 0.036 | 0.001 | 205 |
|  |  | Inferior temporal gyrus | right | BA 37 | 51 | -60 | -9 | 3.99 | 0.201 | 0.008 | 127 |
|  |  | Middle frontal gyrus | left | BA 45 | -42 | 45 | 21 | 3.64 | 0.091 | 0.003 | 162 |
|  |  | Temporal lobe, sub-gyral | right | BA 48 | 27 | -30 | 27 | 3.48 | 0.978 | 0.134 | 34 |
|  |  | Cingulate gyrus | left |  | -9 | 3 | 33 | 3.17 | 0.996 | 0.194 | 25 |
|  |  | Cerebellum | right |  | 33 | -66 | -36 | 3.08 | 0.984 | 0.145 | 32 |
| C > I | fdr 0.05 | Superior frontal gyrus | right | BA 8 | 24 | 30 | 54 | 5.00 | 0.006 | 0.001 | 92 |
|  |  | Superior frontal gyrus | left | BA 8 | -18 | 27 | 51 | 4.90 | 0.006 | 0.001 | 93 |
|  |  | Posterior cingulate gyrus | left | BA 29 | -12 | -48 | 6 | 4.77 | 0.064 | 0.012 | 48 |
|  |  | Posterior cingulate gyrus | right | BA 29 | 12 | -45 | 6 | 4.57 | 0.016 | 0.003 | 72 |
|  |  | Fusiform gyrus | right | BA 20 | 33 | -27 | -18 | 4.32 | 0.040 | 0.007 | 56 |
|  |  | Angular gyrus | left | BA 39 | -45 | -66 | 42 | 4.20 | 0.005 | 0.001 | 96 |
|  |  | Angular gyrus | right | BA 39 | 45 | -69 | 42 | 4.17 | 0.036 | 0.006 | 58 |
|  |  | Medial frontal gyrus | right | BA 11 | 6 | 27 | -9 | 3.98 | 0.223 | 0.045 | 28 |
| C < I | fdr 0.05 | Precentral gyrus | right | BA 6 | 30 | -9 | 51 | 5.25 | 0.001 | 0.000 | 112 |
|  |  | Supplementary Motor Area | left | BA 6 | -15 | -9 | 66 | 4.64 | 0.001 | 0.000 | 127 |
|  |  | Middle occipital gyrus | left | BA 19 | -39 | -84 | -3 | 4.30 | 0.018 | 0.004 | 62 |
|  |  | Superior parietal lobe | left | BA 7 | -18 | -63 | 57 | 4.17 | 0.007 | 0.002 | 78 |
|  |  | Inferior occipital gyrus | right | BA 19 | 45 | -75 | -9 | 4.13 | 0.043 | 0.010 | 48 |
| adolescents > adults (sw − rp) | fdr 0.05 | no suprathreshold clusters | | | | | | | | | |
|  | unc. 0.01 | Middle occipital gyrus | left | BA 19 | -21 | -87 | 21 | 3.49 | 0.000 | 0.000 | 737 |
|  |  | Fusiform gyrus | left | BA 19 | -33 | -75 | -15 | 3.43 | 0.397 | 0.014 | 129 |
| adolescents < adults (sw − rp) | fdr 0.05, unc. 0.01 | no suprathreshold clusters | | | | | | | | | |
| adolescents > adults (I − C) | fdr 0.05 | no suprathreshold clusters | | | | | | | | | |
|  | unc. 0.01 | Inferior frontal gyrus | right | BA 47 | 27 | 33 | 3 | 4.29 | 0.290 | 0.010 | 147 |
| adolescents < adults (I − C) | fdr 0.05, unc. 0.01 | no suprathreshold clusters | | | | | | | | | |
| interaction sw>rp & I> C | fdr 0.05 | Hippocampus | right |  | 30 | -21 | -9 | 4.97 | 0.006 | 0.012 | 25 |
| adolescents > adults (interaction sw>rp & I>C) | fdr 0.05, unc. 0.01 | no suprathreshold clusters | | | | | | | | | |
| adolescents < adults (interaction sw>rp & I>C) | fdr 0.05 | no suprathreshold clusters | | | | | | | | | |
|  | unc. 0.01 | Cingulate cortex | right |  | 12 | -6 | 45 | 3.87 | 0.311 | 0.010 | 143 |

If there were no significant differences at the corrected threshold, we additionally report results from the exploratory analysis (p < 0.01, uncorrected, voxel-level, and p < 0.05, uncorrected, cluster-level, i.e. *k* > 79 voxels). The following abbreviations are used: repeat (rp), switch (sw), congruent trial (C), incongruent trial (I).
