# Supplementary material for: Interindividual Differences in Mid-Adolescents in Error Monitoring and Post-Error Adjustment
Source: PLoS One. 2014 Feb 18;9(2):e88957. doi: 10.1371/journal.pone.0088957 (PMC3928333; doi:10.1371/journal.pone.0088957)
Supplement: Table S3 — Increase in brain response for the second group statistic concerning error trials (N = 181 adolescents and N = 22 adults). If there were no significant differences at the corrected threshold, we additionally report results from the exploratory analysis (p<0.01, uncorrected, voxel-level, and p<0.05, uncorrected, cluster-level, i.e. k >67 voxels). The following abbreviations are used: overall error rate (ER). (DOCX) [file pone.0088957.s003.docx]

Supplementary Table S3: Increase in brain response for the second group statistic concerning error trials (N=181 adolescents and N=22 adults).

| Contrast | p value (voxel-level) | Primary peak location | Hemisphere | Brodmann’s areas | MNI coordinates | | | t | Cluster p (cor.) | Cluster p (unc.) | Cluster size (voxels) |
| --- | --- | --- | --- | --- | --- | --- | --- | --- | --- | --- | --- |
|  |  |  |  |  | x | y | z |  |  |  |  |
| error > correct | fdr 0.05 | no suprathreshold clusters | | | | | | | | | |
|  | unc. 0.01 | Inferior frontal gyrus/Insula | left | BA 47 | -33 | 18 | -6 | 4.92 | 0.029 | 0.001 | 247 |
|  |  | Inferior frontal gyrus | right |  | 33 | 24 | -6 | 4.80 | 0.029 | 0.001 | 247 |
|  |  | Medial frontal gyrus | right | BA 32 | 9 | 21 | 48 | 3.92 | 0.000 | 0.000 | 592 |
|  |  | Midbrain | left |  | -6 | -24 | -3 | 3.47 | 0.258 | 0.008 | 138 |
|  |  | Inferior parietal lobe | left | BA 40 | -51 | -39 | 48 | 3.22 | 0.623 | 0.025 | 91 |
|  |  | Inferior parietal lobe | right | BA 40 | 45 | -48 | 48 | 3.16 | 0.143 | 0.004 | 167 |
|  |  | Middle frontal gyrus | right | BA 6 | 39 | 0 | 54 | 3.11 | 0.464 | 0.016 | 108 |
| adolescents > adults | fdr 0.05, unc. 0.01 | no suprathreshold clusters | | | | | | | | | |
| adolescents < adults | fdr 0.05, unc. 0.01 | no suprathreshold clusters | | | | | | | | | |
| ER correlates pos. adolescents | fdr 0.05 | no suprathreshold clusters | | | | | | | | | |
|  | unc. 0.01 | Occipital lobe, cuneus | right | BA 19 | 15 | -81 | 21 | 3.90 | 0.263 | 0.008 | 137 |
|  |  | Supramarginal gyrus | left | BA 39 | -36 | -57 | 27 | 3.77 | 0.613 | 0.024 | 92 |
|  |  | Paracentral lobule | right | BA 4 | 6 | -36 | 69 | 3.56 | 0.069 | 0.002 | 203 |
|  |  | Cerebellum | left |  | -33 | -81 | -24 | 3.40 | 0.792 | 0.040 | 74 |
| ER correlates pos. adults | fdr 0.05, unc. 0.01 | no suprathreshold clusters | | | | | | | | | |
| ER correlates neg. adolescents | fdr 0.05 | Inferior frontal gyrus/Insula | left | BA 47 | -33 | 21 | -6 | 5.33 | 0.000 | 0.000 | 63 |
|  |  | Inferior frontal gyrus/Insula | right | BA 47 | 33 | 24 | 0 | 4.40 | 0.008 | 0.006 | 32 |
| ER correlates neg. adults | fdr 0.05, unc. 0.01 | no suprathreshold clusters | | | | | | | | | |
| adolescents > adults (interaction ER) | fdr 0.05, unc. 0.01 | no suprathreshold clusters | | | | | | | | | |
| adolescents < adults (interaction ER) | fdr 0.05, unc. 0.01 | no suprathreshold clusters | | | | | | | | | |

If there were no significant differences at the corrected threshold, we additionally report results from the exploratory analysis (p < 0.01, uncorrected, voxel-level, and p < 0.05, uncorrected, cluster-level, i.e. *k* > 67 voxels). The following abbreviations are used: overall error rate (ER).
