# Supplementary material for: Interindividual Differences in Mid-Adolescents in Error Monitoring and Post-Error Adjustment
Source: PLoS One. 2014 Feb 18;9(2):e88957. doi: 10.1371/journal.pone.0088957 (PMC3928333; doi:10.1371/journal.pone.0088957)
Supplement: Table S4 — Increase in brain response for the third group statistic concerning correct trials following errors or missing trials (N = 181 adolescents and N = 22 adults). If there were no significant differences at the corrected threshold, we additionally report results from the exploratory analysis (p<0.01, uncorrected, voxel-level, and p<0.05, uncorrected, cluster-level, i.e. k >75 voxels). The following abbreviations are used: overall error rate (ER), supplementary motor area (SMA). (DOCX) [file pone.0088957.s004.docx]

Supplementary Table S4: Increase in brain response for the third group statistic concerning correct trials following errors or missing trials (N=181 adolescents and N=22 adults).

| Contrast | p value (voxel-level) | Primary peak location | Hemisphere | Brodmann’s areas | MNI coordinates | | | t | Cluster p (cor.) | Cluster p (unc.) | Cluster size (voxels) |
| --- | --- | --- | --- | --- | --- | --- | --- | --- | --- | --- | --- |
|  |  |  |  |  | x | y | z |  |  |  |  |
| post-error > correct | fdr 0.05 | Superior frontal gyrus | right | BA 8 | 6 | 27 | 63 | 5.87 | 0.000 | 0.000 | 2801 |
|  |  | Angular gyrus | right | BA 40 | 60 | -48 | 36 | 5.10 | 0.000 | 0.000 | 486 |
|  |  | Cerebellum | left |  | -33 | -81 | -27 | 5.06 | 0.000 | 0.000 | 877 |
|  |  | Inferior frontal gyrus | left |  | -42 | 18 | -12 | 4.72 | 0.011 | 0.000 | 208 |
|  |  | Inferior parietal lobe | left | BA 39 | -54 | -54 | 42 | 4.12 | 0.003 | 0.000 | 265 |
|  |  | Middle temporal gyrus | right | BA 21 | 69 | -36 | 0 | 3.95 | 0.025 | 0.001 | 176 |
|  |  | Posterior cingulate cortex | right | BA 23 | 3 | -21 | 39 | 3.92 | 0.072 | 0.003 | 136 |
|  |  | Thalamus | left |  | -12 | -12 | 0 | 3.70 | 0.129 | 0.006 | 115 |
|  |  | Middle frontal gyrus | left | BA 44 | -39 | 21 | 39 | 3.48 | 0.033 | 0.001 | 165 |
|  |  | Occipital lobe, lingual gyrus | left | BA 18 | -21 | -96 | -12 | 3.34 | 0.719 | 0.051 | 50 |
|  |  | Middle temporal gyrus | left | BA 20 | -51 | -33 | -9 | 3.14 | 0.969 | 0.138 | 27 |
|  |  | Thalamus | right |  | 12 | -6 | 3 | 3.02 | 0.969 | 0.138 | 27 |
| adolescents > adults | fdr 0.05, unc. 0.01 | no suprathreshold clusters | | | | | | | | | |
| adolescents < adults | fdr 0.05 | no suprathreshold clusters | | | | | | | | | |
|  | unc. 0.01 | Middle frontal gyrus | left | BA 10 | -30 | 60 | 15 | 3.38 | 0.691 | 0.031 | 87 |
| ER correlates pos. adolescents | fdr 0.05 | no suprathreshold clusters | | | | | | | | | |
|  | unc. 0.01 | Corpus callosum | left |  | -15 | 27 | 9 | 3.60 | 0.353 | 0.012 | 127 |
| ER correlates pos. adults | fdr 0.05, unc. 0.01 | no suprathreshold clusters | | | | | | | | | |
| ER correlates neg. adolescents | fdr 0.05 | Inferior parietal lobe | right | BA 39 | 54 | -54 | 42 | 5.44 | 0.000 | 0.000 | 646 |
|  |  | Angular gyrus | left | BA 39 | -54 | -54 | 33 | 5.17 | 0.000 | 0.000 | 391 |
|  |  | Superior frontal gyrus/SMA | right | BA 8 | 12 | 21 | 63 | 5.07 | 0.000 | 0.000 | 1119 |
|  |  | Middle frontal gyrus | right | BA 9 | 42 | 15 | 48 | 5.01 | 0.003 | 0.000 | 201 |
|  |  | Middle temporal gyrus | right | BA 20 | 63 | -30 | -12 | 4.55 | 0.012 | 0.001 | 156 |
|  |  | Middle frontal gyrus | left | BA 9 | -36 | 9 | 51 | 4.45 | 0.022 | 0.001 | 138 |
|  |  | Posterior cingulate cortex | right | BA 23 | 3 | -21 | 39 | 4.40 | 0.004 | 0.000 | 197 |
|  |  | Middle temporal gyrus | left | BA 21 | -66 | -27 | -9 | 3.77 | 0.434 | 0.031 | 51 |
|  |  | Inferior occipital gyrus | right | BA 18 | 30 | -90 | 0 | 3.69 | 0.130 | 0.008 | 85 |
|  |  | Inferior occipital gyrus | left | BA 18 | -18 | -93 | -9 | 3.56 | 0.652 | 0.058 | 38 |
|  |  | Inferior frontal gyrus | left | BA 38 | -45 | 18 | -9 | 3.40 | 0.529 | 0.041 | 45 |
|  |  | Cerebellum | left |  | -36 | -72 | -33 | 3.39 | 0.849 | 0.104 | 27 |
|  |  | Middle frontal gyrus | left | BA 46 | -42 | 54 | 9 | 3.23 | 0.864 | 0.110 | 26 |
|  |  | Precuneus | right |  | 12 | -54 | 39 | 3.23 | 0.879 | 0.116 | 25 |
| ER correlates neg. adults | fdr 0.05, unc. 0.01 | no suprathreshold clusters | | | | | | | | | |
| adolescents > adults (interaction ER) | fdr 0.05, unc. 0.01 | no suprathreshold clusters | | | | | | | | | |
| adolescents < adults (interaction ER) | fdr 0.05 | no suprathreshold clusters | | | | | | | | | |
|  | unc. 0.01 | Superior frontal gyrus/SMA | left | BA 6 | -9 | 18 | 63 | 4.01 | 0.401 | 0.014 | 120 |

If there were no significant differences at the corrected threshold, we additionally report results from the exploratory analysis (p < 0.01, uncorrected, voxel-level, and p < 0.05, uncorrected, cluster-level, i.e. *k* > 75 voxels). The following abbreviations are used: overall error rate (ER), supplementary motor area (SMA).
